# Maternal High-Sucrose Diet Affects Phenotype Outcome in Adult Male Offspring: Role of *Zbtb16*

**DOI:** 10.3389/fgene.2020.529421

**Published:** 2020-09-11

**Authors:** Elena Školníková, Lucie Šedová, Blanka Chylíková, Adéla Kábelová, František Liška, Ondřej Šeda

**Affiliations:** Institute of Biology and Medical Genetics, First Faculty of Medicine, Charles University and General University Hospital, Prague, Czechia

**Keywords:** DOHAD, Zbtb16, maternal nutrition, transcriptomics, high-sucrose diet

## Abstract

Overnutrition in pregnancy and lactation affects fetal and early postnatal development, which can result in metabolic disorders in adulthood. We tested a hypothesis that variation of the *Zbtb16* gene, a significant energy metabolism regulator, modulates the effect of maternal high-sucrose diet (HSD) on metabolic and transcriptomic profiles of the offspring. We used the spontaneously hypertensive rat (SHR) strain and a minimal congenic rat strain SHR-*Zbtb16*, carrying the *Zbtb16* gene allele originating from the PD/Cub rat, a metabolic syndrome model. Sixteen-week-old SHR and SHR-*Zbtb16* rat dams were fed either standard diet (control groups) or a high-sucrose diet (HSD, 70% calories as sucrose) during pregnancy and 4 weeks of lactation. In dams of both strains, we observed an HSD-induced increase of cholesterol and triacylglycerol concentrations in VLDL particles and a decrease of cholesterol and triacylglycerols content in medium to very small LDL particles. In male offspring, exposure to maternal HSD substantially increased brown fat weight in both strains, decreased triglycerides in LDL particles, and impaired glucose tolerance exclusively in SHR. The transcriptome assessment revealed networks of transcripts reflecting the shifts induced by maternal HSD with major nodes including *mir-126, Hsd11b1* in the brown adipose tissue, *Pcsk9, Nr0b2* in the liver and *Hsd11b1, Slc2a4* in white adipose tissue. In summary, maternal HSD feeding during pregnancy and lactation affected brown fat deposition and lipid metabolism in adult male offspring and induced major transcriptome shifts in liver, white, and brown adipose tissues. The *Zbtb16* variation present in the SHR-*Zbtb16* led to several strain-specific effects of the maternal HSD, particularly the transcriptomic profile shifts of the adult male offspring.

## Introduction

Overconsumption of high-carbohydrate, high-fat foods is a dietary pattern typical for Western societies and has been gaining ground also in populations that were previously used to a mostly plant-based diet. It has been linked to the development of obesity, dyslipidemia, and type 2 diabetes (Softic et al., 2017), the components of metabolic syndrome (Alberti et al., 2009). As the prevalence of these ailments and their related complications has globally risen over the last few decades, there is a growing need for understanding of their contributing genetic and environmental factors. Although genome-wide association studies have identified numerous DNA variants contributing to the genetic architecture of type 2 diabetes, dyslipidemia, or obesity, the explanation of the increase in their prevalence is still incomplete (Barroso and McCarthy, 2019). Exposure to environmental stimuli during early stages of development, characterized by rapid cell proliferation/differentiation, can establish permanent changes in offspring physiology or metabolism via a process dubbed developmental programming (Fleming et al., 2018). Developmental origins of health and disease (DOHAD) hypothesis proposes a way of how nutrition may influence later health, if acting during a critical window of development, and has been supported by animal studies as well as evidence in humans (Langley-Evans, 2009; Nicholas and Ozanne, 2019). Inappropriate nutrition in pregnancy may be considered as a maternal nutritional stress and can program offspring metabolism (Arentson-Lantz et al., 2016). Several studies have investigated the effects of maternal overnutrition, mostly induced by high-fat, or high-carbohydrate, high-fat diets (Nicholas and Ozanne, 2019), but only a few of the studies concentrated on the effects of carbohydrates in particular (Sedova et al., 2007; D’Alessandro et al., 2014; Skolnikova et al., 2020). Also, there is only limited data on the potential effect of genetic factors in the determination of susceptibility to nutritional programming. One of the genes potentially interconnecting all major components of the metabolic syndrome is zinc finger and BTB domain containing 16 (*ZBTB16*) transcription factor (Seda et al., 2017). In addition to its role in adipogenesis, control of hepatic gluconeogenesis (Chen et al., 2014), and cardiac hypertrophy (Liska et al., 2017), genetic variation in human ZBTB16 was associated with increased obesity parameters and higher total and LDL cholesterol (Bendlova et al., 2017). We have previously shown that rats carrying specific *Zbtb16* allele are particularly sensitive to HSD, glucocorticoids, and retinoic acid in terms of induction of metabolic dysfunction (Krupkova et al., 2014, 2018). Therefore, we hypothesized that maternal HSD intake during pregnancy and lactation would program the offspring to develop metabolic alterations in adulthood and that these effects may be, to a certain extent, modified by genetic variation in *Zbtb16*. We used two inbred rat strains: the spontaneously hypertensive rat (SHR), widely used model of essential hypertension prone to develop lipid and glucose metabolism disturbances (Pravenec et al., 2014), and the newly derived minimal congenic strain SHR-*Zbtb16* differing from SHR by a 254 kb differential segment of rat chromosome 8 containing only a mutated variant of *Zbtb16* gene of the PD/Cub strain origin (Liska et al., 2009).

## Materials and Methods

### Ethical Statement

All experiments were performed in agreement with the Animal Protection Law of the Czech Republic (311/1997) which is in compliance with the European Community Council recommendations for the use of laboratory animals 86/609/ECC and were approved by the Ethical Committee of the First Faculty of Medicine of the Charles University and by the Ministry of Education, Youth and Sports (protocol no. MSMT-14076/2015-14).

### Rat Strains

The spontaneously hypertensive rat (SHR/OlaIpcv, Rat Genome Database (RGD) ID 631848) (Shimoyama et al., 2017) as a commonly used model of essential hypertension was used because of its known metabolic abnormalities (Langley et al., 2013). The SHR-*Lx*.PD5^PD–^*^Zbtb16^* single congenic strain (SHR-*Zbtb16* hereafter) carries the *Zbtb16* gene of polydactylous rat (PD/Cub, RGD ID 728161) origin on the SHR genomic background. The derivation of this strain was described previously (Seda et al., 2005; Krupkova et al., 2018). Both strains are highly inbred and maintained by brother x sister mating at the Institute of Biology and Medical Genetics.

### Experimental Protocol

Animals were held under temperature and humidity-controlled conditions on 12 h/12 h light-dark cycle. At all times, the animals had free access to food and water.

#### Rat Dams – Experimental Protocol

**FIGURE 1:**
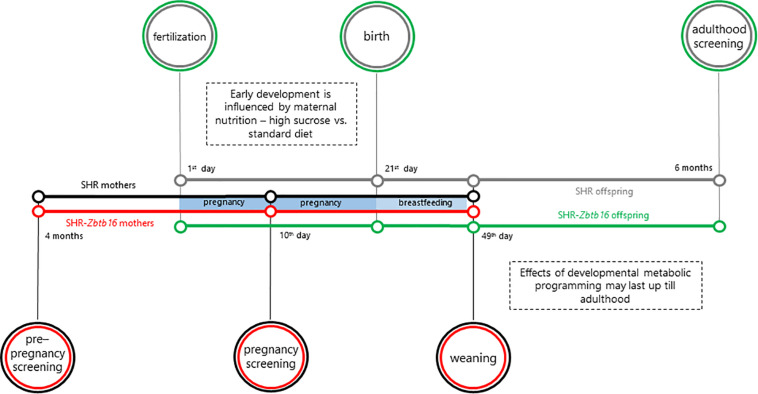
Outline of the study. The colors of the timelines indicate the individual groups: SHR rat dams (mothers, black), SHR-*Zbtb16* rat dams (mothers, red), SHR offspring (gray), SHR-*Zbtb16* offspring (green).

As outlined in Figure 1, 16-week-old female rat controls of SHR and SHR-*Zbtb16* strains (*n* = 6/strain) were fed a standard diet (STD, ssniff RZ, ssniff Spezialdiäten GmbH, Soest, Germany) during pregnancy and 4 weeks of lactation. Experimental groups of SHR and SHR-*Zbtb16* rat dams (*n* = 6/strain) were fed a high-sucrose diet [HSD, proteins (19.6 cal%), fat (10.4 cal%), carbohydrates (sucrose, 70 cal%)] in the same period. The diets contained equal amounts of micronutrients and vitamins (Supplementary Material). Blood samples for metabolic and glycemic assessments were drawn after overnight fasting from the tail vein. The OGTT of females was performed at 16 weeks of age and on the 10th day of pregnancy. The blood samples were obtained at intervals of 0, 30, 60, 120, and 180 min after intragastric glucose administration to conscious rats (3 g/kg body weight, 30% aqueous solution; Ascensia Elite Blood Glucose Meter; Bayer HealthCare, Mishawaka, IN, United States; validated by the Institute of Clinical Biochemistry and Laboratory Diagnostics of the First Faculty of Medicine).

#### Rat Dams – Serum Phenotyping

Metabolic profile of rat dams was assessed via 13-plex Milliplex^®^ MAP Rat Metabolic Hormone Magnetic Bead Panel Kit using BioPlex system (Bio-Rad), (Merck Millipore Corp., Billerica, MA, United States) for levels of amylin, C-peptide 2, ghrelin, GIP, GLP-1, glucagon, IL-6, insulin, leptin, MCP-1, PP. PYY and TNFα. Cytokine profile of rat dams was assessed via Bio-Plex Pro Rat Cytokine 24-Plex Panel (Bio-Rad Laboratories, Inc., Luminex Corporation) for levels of EPO, G-CSF, GM-CSF, GRO/KC, IFN-γ, IL-1α, IL-1β, IL-2, IL-4, IL-5, IL-6, IL-7, IL-10, IL-12p70, IL-13, IL-17, IL-18, M-CSF, MCP-1, MIP-1α, MIP-3α, RANTES, TNF-α, VEGF using BioPlex system (Bio-Rad). The lipid profile was assessed using high-performance liquid chromatography (HPLC) for determining triacylglycerol and cholesterol concentrations in 20 lipoprotein fractions and the size of major classes of lipoprotein particles as described previously (Usui et al., 2002).

#### The Breeding Protocol and Offspring Phenotyping

The SHR and SHR-*Zbtb16* rat dams were bred with the corresponding (SHRxSHR, SHR-*Zbtb16* x SHR-*Zbtb16*) males, i.e., only homozygous, inbred SHR and inbred SHR-*Zbtb16* animals were therefore used throughout the experiment. The litter size was restricted to eight pups both in SHR and SHR-*Zbtb16* offspring, which were weaned after 28 days and fed standard diet till the age of 6 months. At that time, SHR and SHR-*Zbtb16* male offspring of both control (*n* = 8/strain, at least one rat per litter) and experimental (*n* = 8/strain, at least one rat per litter) groups were subjected to OGTT, blood draw for metabolic and lipid profile assessment and sacrificed to determine the weights of heart, liver, kidneys, adrenals, interscapular brown fat, epididymal and retroperitoneal fat pads. Liver, interscapular brown fat, and epididymal adipose tissue were snap-frozen in liquid nitrogen in preparation for the transcriptome assessment.

### Microarrays – Transcriptome Assessment

Total RNA was isolated from liver tissue (RNeasy Mini Kit, Qiagen), epididymal (visceral), and brown fat (RNeasy Lipid Tissue Mini Kit, Qiagen). The quality and integrity of the total RNA was evaluated on Agilent 2100 Bioanalyzer system (Agilent, Palo Alto, CA). Microarray experiments were performed using the Rat Gene 2.1 ST Array Strip in quadruplicate (per strain/programming). The whole hybridization procedure was performed using the Affymetrix GeneAtlas^®^ system according to the manufacturer’s instructions. The quality control of the chips was performed using Affymetrix Expression Console. Partek Genomics Suite (Partek, St. Louis, Missouri) was used for subsequent data analysis. After applying quality filters and data normalization by Robust Multichip Average (RMA) algorithm implemented in Affymetrix Expression Console, the set of obtained differentially expressed probesets was filtered by false discovery rate (FDR) method implemented in PARTEK Genomics Suite 6.6 (Partek, St. Louis, Missouri). Transcriptomic data were then processed by a standardized sequence of analyses (hierarchical clustering and principal component analysis, gene ontology, gene set enrichment, “Upstream Regulator Analysis,” “Mechanistic Networks,” “Causal Network Analysis,” and “Downstream Effects Analysis”) using Ingenuity Pathway Analysis.

### RT-qPCR

To validate microarray gene expression data, we performed quantitative real-time PCR (RT-qPCR) using TaqMan^®^ probes (Applied Biosystems^TM^) according to the manufacturer’s instruction. Total RNA (1 μg) was reverse-transcribed with oligo-dT primers using the SuperScript III (Invitrogen). Real-time PCR reaction was performed in triplicate with TaqMan^®^ Gene Expression Master Mix (Applied Biosystems) according to the manufacturer’s protocol (Invitrogen) using Applied Biosystems^®^ 7900HT Real-Time PCR System. Results were analyzed using the Livak analysis method (Livak and Schmittgen, 2001) with glyceraldehyde 3-phosphate dehydrogenase (*Gapdh*) as reference gene. The probes used for validation were Rn00567167_m1, Rn00562884_m1, Rn00566938_m1, Rn00581867_m1, Rn00593680_m1, Rn00589173_m1, Rn01416753_m1, Rn01495769_m1, Rn01789864_s1, Rn00567532_m1, Rn00562597_m1, Rn01454585_g1, Rn00567668_m1.

### Statistical Analysis

All statistical analyses were performed using STATISTICA 13.5 (TIBCO). When comparing morphometric and biochemical variables between groups, two-way ANOVA with STRAIN and DIET as major factors were used, followed by *post-hoc* Fisher’s test for comparison of the specific pairs of variables. Glucose concentration data during OGTT were analyzed by repeated-measures ANOVA. Benjamini-Hochberg procedure was applied to control the false discovery rate (Benjamini and Hochberg, 1995). Null hypothesis was rejected whenever *p* >0.05.

### Data Availability

The datasets generated during and/or analyzed during the current study are available from the corresponding author on reasonable request. The microarray data generated during and/or analyzed during the current study are available in the ArrayExpress repository, Experiment ArrayExpress accession: E-MTAB-6838^1^.

## Results

### Body Weight and Food Intake in Rat Dams

**FIGURE 2:**
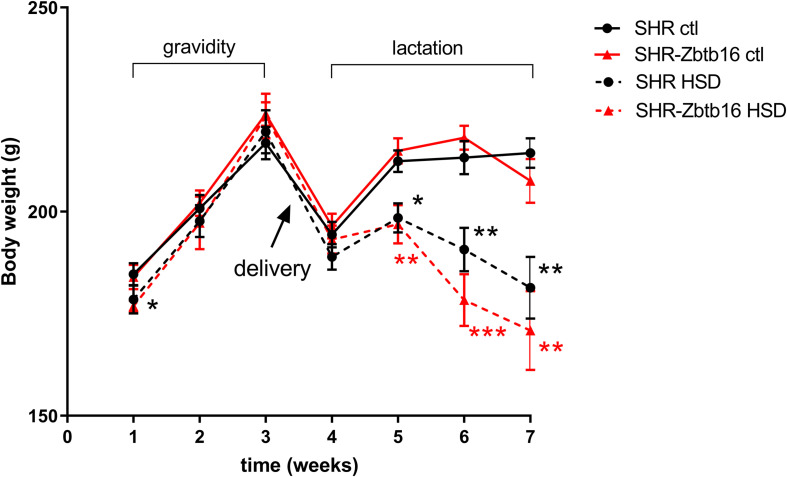
Bodyweight measurements of SHR (black circles) and SHR-*Zbtb16* (red triangles) adult female rats in gravidity (weeks 1–3) and lactation period (weeks 4–7): SHR control females (SHR ctl – full black line), SHR females fed HSD (SHR HSD – dashed black line), SHR-*Zbtb16* control females (SHR-*Zbtb16* ctl – full red line), SHR-*Zbtb16* females fed HSD (SHR-*Zbtb16* HSD – dashed red line). Data are expressed as mean ± SEM. Within the graph, the significance levels of repeated-measures ANOVA for HSD-fed vs. control females within each strain are indicated as follows: *p < 0.05, **p < 0.01, ***p < 0.001.

Before gravidity, SHR and SHR-*Zbtb16* females showed similar weight (Figure 2), overall glucose tolerance, and distribution of triacylglycerols (TG) and cholesterol into lipoprotein fractions (Supplementary Material). As the amount of consumed diet was not significantly different between the HSD and STD-fed groups (HSD SHR: 39 ± 2 g/day, HSD SHR-*Zbtb16*: 43 ± 2 g/day, STD SHR: 43 ± 3 g/day and STD SHR-*Zbtb16*: 47 ± 4 g/day), the estimated metabolizable energy intake was also comparable among the groups. HSD-fed rat dams of both strains showed a subsequent significant decrease in body weight compared to STD-fed controls during the period of breastfeeding despite keeping comparable energy intake to the control groups (Figure 2).

### Metabolic Profile of Rat Dams

**FIGURE 3:**
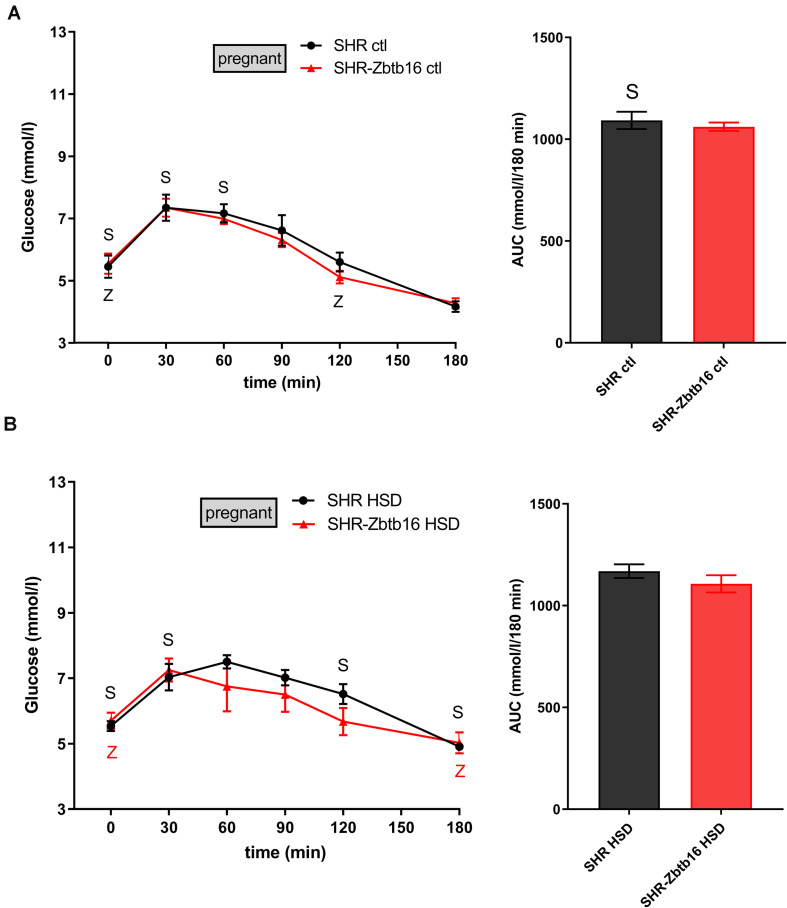
The oral glucose tolerance test (OGTT). The course of glycemic curves in pregnant SHR females (black circles) and pregnant SHR-*Zbtb16* females (red triangles) fed STD (ctl, **A**) or fed HSD (HSD, **B**) during OGTT with corresponding areas under the curves (AUC). Data are expressed as mean ± SEM. OGTT before pregnancy is shown in Supplementary Material. The significance levels of pair-wise comparisons (control before pregnancy vs. control in pregnancy vs. HSD fed in pregnancy) by repeated-measures two-way ANOVA (OGTT) and *post-hoc* Fisher’s test of the two-way ANOVA (AUC) with STRAIN and DIET as major factors are indicated as follows: ^∗^*p* < 0.05, ^∗∗^*p* < 0.01, ^∗∗∗^*p* < 0.001. **S** represents differences in SHR – **(A)** *t* = 0 min^∗^, *t* = 30 min^∗^, *t* = 60 min^∗^, AUC^∗^, between control females before and during pregnancy; **(B)** *t* = 0 min^∗∗^, *t* = 30 min^∗∗^, *t* = 120 min^∗^, *t* = 180 min^∗∗^, between control females before pregnancy and females fed HSD during pregnancy. **Z** represents differences in SHR-*Zbtb16* – panel **(A)** *t* = 0 min^∗∗^, *t* = 120 min^∗^ between control females before and during pregnancy; **(B)** *t* = 0 min^∗∗^, *t* = 180 min^∗^ between control females before pregnancy and females fed HSD during pregnancy.

**FIGURE 4:**
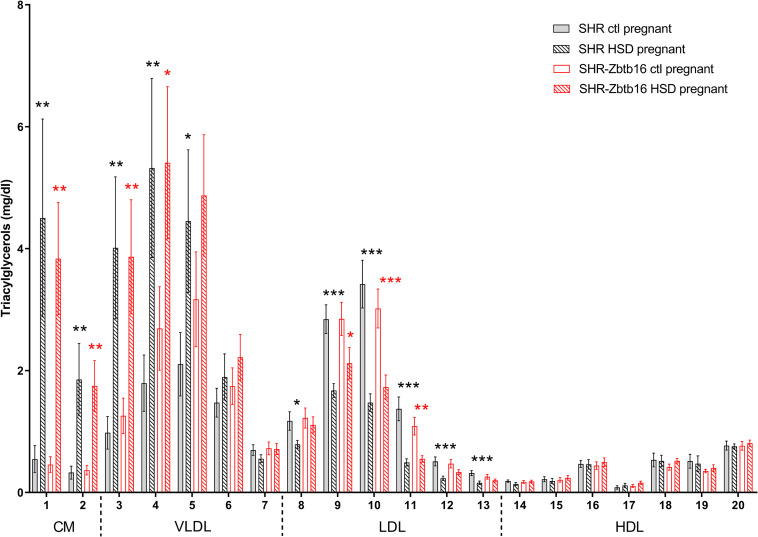
Triacylglycerol profile. The triacylglycerol (Tg) content in 20 lipoprotein subfractions in pregnant SHR female rats and SHR-*Zbtb16* female rats – SHR fed standard diet in pregnancy (SHR ctl pregnant – light gray bars), SHR fed HSD in pregnancy (SHR HSD pregnant – black patterned bars), SHR-*Zbtb16* fed standard diet in pregnancy (SHR-*Zbtb16* ctl pregnant – open red bars) and SHR-*Zbtb16* fed HSD in pregnancy (SHR-*Zbtb16* HSD pregnant – patterned red bars). Tg profile before pregnancy is shown in Supplementary Material. Within the graph and this legend, the significance levels of pair-wise comparisons (ctl pregnant vs. HSD pregnant) by *post-hoc* Fisher’s test of the two-way ANOVA with STRAIN and DIET as major factors are indicated as follows: *p < 0.05, **p < 0.01, ***p < 0.001. Black asterisks mark significance levels between SHR ctl pregnant and SHR HSD pregnant, red asterisks mark significance levels between SHR-*Zbtb16* ctl pregnant and SHR-*Zbtb16* HSD pregnant. The allocation of individual lipoprotein subfractions to major lipoprotein classes is shown in order of particle’s decreasing size from left to right. CM, chylomicron; VLDL, very low density lipoprotein; LDL, low density lipoprotein; HDL, high density lipoprotein.

**FIGURE 5:**
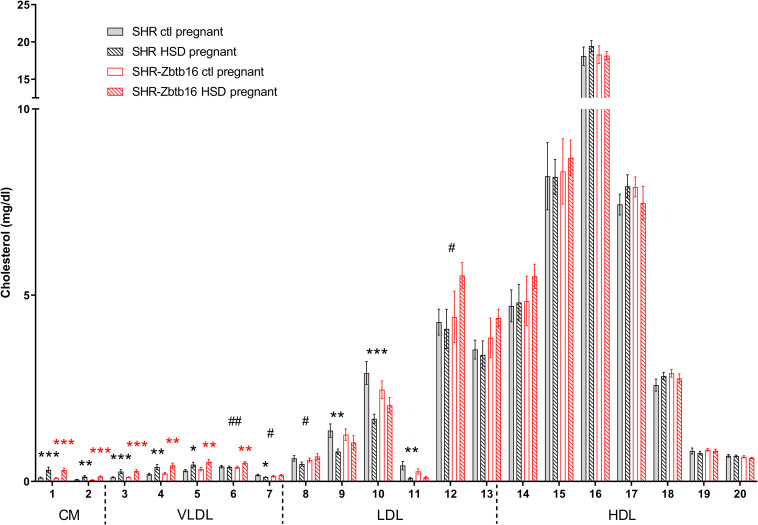
Cholesterol profile. The cholesterol (C) content in 20 lipoprotein subfractions in pregnant SHR female rats and SHR-*Zbtb16* female rats – SHR fed standard diet in pregnancy (SHR ctl pregnant – light gray bars), SHR fed HSD in pregnancy (SHR HSD pregnant – black patterned bars), SHR-*Zbtb16* fed standard diet in pregnancy (SHR-*Zbtb16* ctl pregnant – open red bars) and SHR-*Zbtb16* fed HSD in pregnancy (SHR-*Zbtb16* HSD pregnant – patterned red bars). Cholesterol profile before pregnancy is shown in Supplementary Material. Within the graph and this legend, the significance levels of pair-wise comparisons (ctl pregnant vs. HSD pregnant) by *post-hoc* Fisher’s test of the two-way ANOVA with STRAIN and DIET as major factors are indicated as follows: *p < 0.05, **p < 0.01, ***p < 0.001. Black asterisks mark significance levels between SHR ctl pregnant and SHR HSD pregnant, red asterisks mark significance levels between SHR-*Zbtb16* ctl pregnant and SHR-*Zbtb16* HSD pregnant. # represents differences in specific lipoprotein fractions between SHR HSD pregnant and SHR-*Zbtb16* HSD pregnant; ^#^*p* < 0.05, ^##^*p* < 0.01. C6^##^, C7^#^, C8^#^, C12^#^, which were all higher in SHR-*Zbtb16*. The allocation of individual lipoprotein subfractions to major lipoprotein classes is shown in order of particle’s decreasing size from left to right. CM, chylomicron; VLDL, very low density lipoprotein; LDL, low density lipoprotein; HDL, high density lipoprotein.

While the SHR-*Zbtb16* females showed better glucose tolerance compared to SHR before pregnancy (Supplementary Material), this difference was not apparent in pregnant rats irrespective of the administered diet (Figure 3). No strain differences were observed for TG distribution into individual lipoprotein classes in pregnant SHR and SHR-*Zbtb16* fed STD or HSD (Figure 4). On the other hand, pregnant HSD SHR-*Zbtb16*dams displayed a higher concentration of cholesterol in medium and small very-low-density lipoproteins (VLDL) as well as in large low-density lipoproteins (LDL) compared to HSD SHR (Figure 5). The assessment of metabolic parameters on the 10th day of gravidity showed significant shifts in lipid profile induced by the pregnancy itself and also by diet in HSD-fed dams. In both strains, we observed increased mid-gravidity concentrations of cholesterol in medium and small VLDL fractions, most of LDL fractions and very large and very small high-density lipoprotein (HDL) fractions compared to pre-pregnancy concentrations (Figure 5). Similarly, corresponding TG fractions followed the same pattern and were increased in both strains compared to pre-pregnancy values (Figure 4). In addition, HSD in pregnancy significantly increased both cholesterol and triacylglycerols content in chylomicron fraction with a slightly more prominent effect in SHR-*Zbtb16* rat dams.

### Cytokine and Hormone Profile of Rat Dams

**FIGURE 6:**
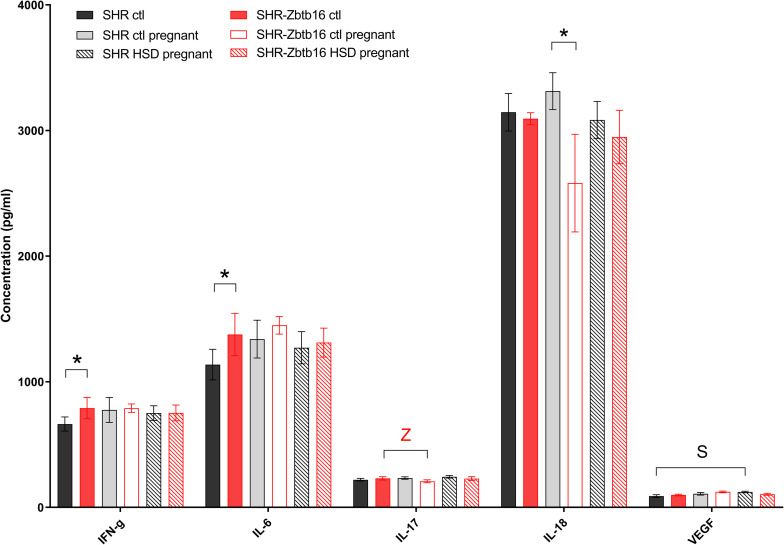
Cytokine profile. Cytokine concentrations in the serum of SHR female rats fed standard diet before pregnancy (SHR ctl: black bars, SHR-*Zbtb16* ctl: red bars), standard diet in pregnancy (SHR ctl pregnant: light gray bars, SHR-*Zbtb16* ctl pregnant: empty red bars), HSD in pregnancy (SHR HSD pregnant: black patterned bars, SHR-*Zbtb16* HSD pregnant: red patterned bars). Within the graph, the significance levels of pair-wise group comparison (ctl vs. ctl pregnant vs. HSD pregnant) by *post-hoc* Fisher’s test of the two-way ANOVA with STRAIN and DIET as major factors are indicated as follows: *p < 0.05. Data are expressed as mean ± SEM. **S** represents differences in SHR: VEGF^∗^ between SHR ctl and SHR HSD pregnant. **Z** represents differences in SHR-*Zbtb16*: IL-17^∗^ between SHR-*Zbtb16* ctl and SHR-*Zbtb16* ctl pregnant.

**FIGURE 7:**
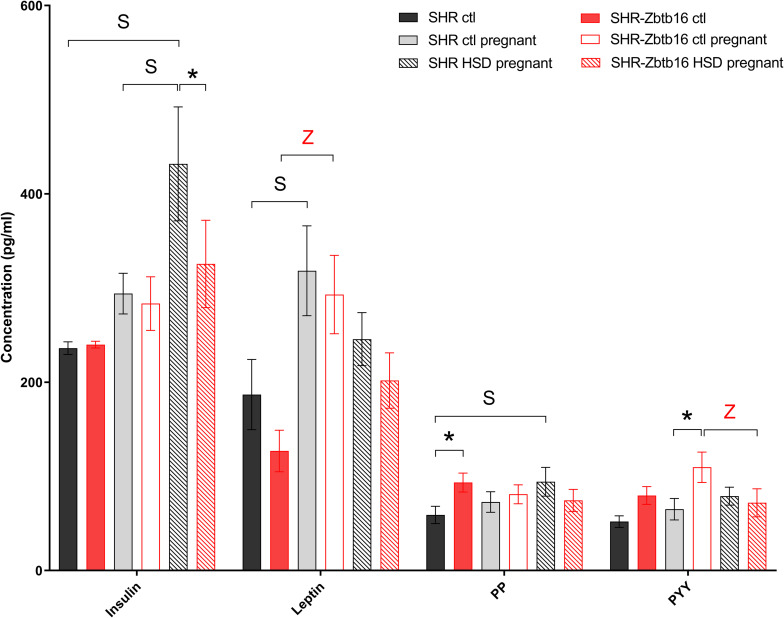
Hormone profile. Hormone concentrations in the serum of female rats fed standard diet before pregnancy (SHR ctl: black bars, SHR-*Zbtb16* ctl: red bars), standard diet in pregnancy (SHR ctl pregnant: light gray bars, SHR-*Zbtb16* ctl pregnant: empty red bars), HSD in pregnancy (SHR HSD pregnant: black patterned bars, SHR-*Zbtb16* HSD pregnant: red patterned bars). Within the graph, the significance levels of pair-wise group comparison (ctl vs. ctl pregnant vs. HSD pregnant) by *post-hoc* Fisher’s test of the two-way ANOVA with STRAIN and DIET as major factors are indicated as follows: *p < 0.05. Data are expressed as mean ± SEM. **S** represents differences in SHR: Leptin^∗^ between SHR ctl and SHR ctl pregnant, PP^∗^ between SHR ctl and SHR HSD pregnant. **Z** represents differences in SHR-*Zbtb16*: Leptin^∗∗^ betweenSHR-*Zbtb16* ctl and SHR-*Zbtb16* ctl pregnant, PYY^∗^ between SHR-*Zbtb16* ctl pregnant and SHR-*Zbtb16* HSD pregnant.

Before pregnancy, the concentrations of cytokines were comparable between the two strains except for levels of interleukin 6, interferon gamma (Figure 6), and pancreatic polypeptide (Figure 7) that were slightly higher in SHR-*Zbtb16* dams. In both strains, the pregnancy increased levels of leptin and, exclusively in SHR-*Zbtb16*, decreased the concentrations of interleukin 17 (Figure 6). The mid-pregnancy interleukin 18 was increased, and polypeptide YY was decreased in SHR compared to SHR-*Zbtb16*. Exposure to HSD in pregnancy led to an elevation of vascular endothelial growth factor and pancreatic polypeptide concentrations only in SHR dams. The insulin level was highest in HSD SHR compared both to HSD SHR-*Zbtb16* and STD SHR dams (Figure 7).

### Morphometric Profile of Adult Male Offspring

**FIGURE 8:**
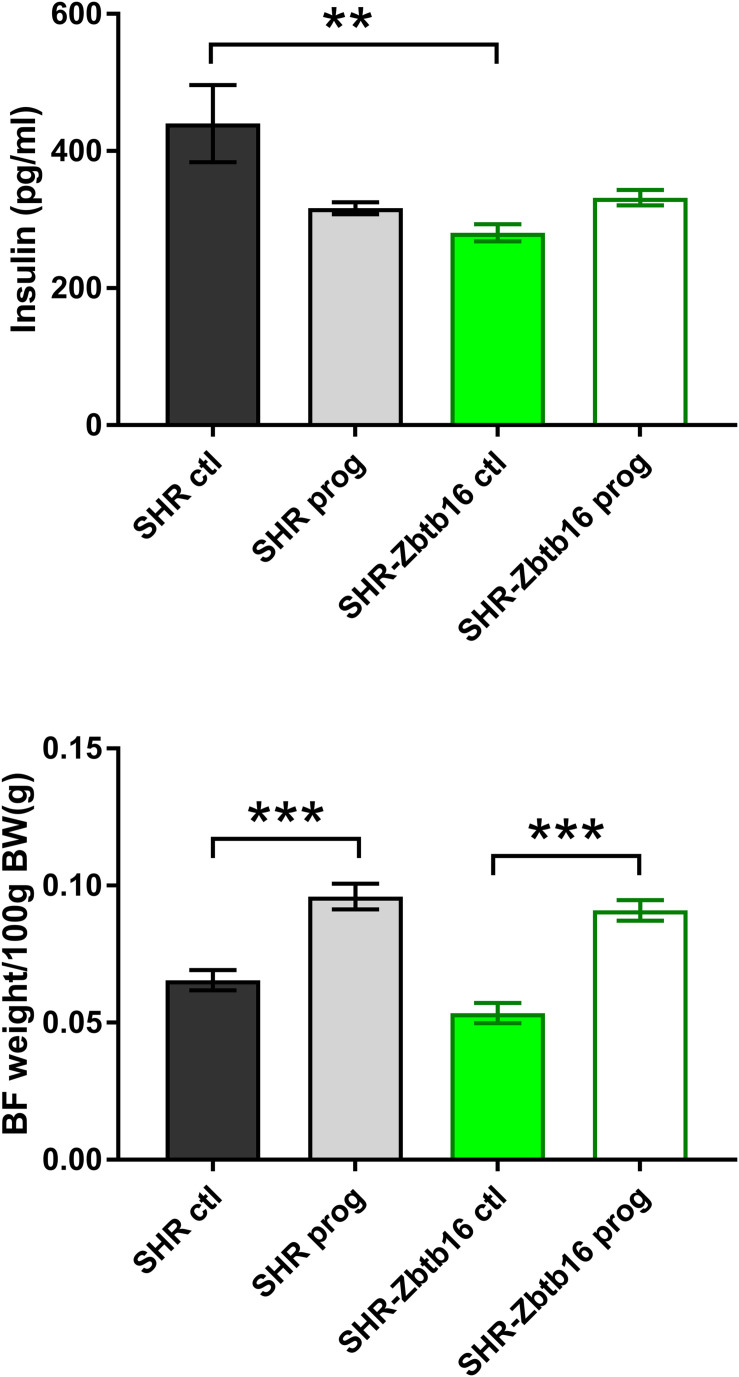
Fasting insulin concentrations (top panel) and interscapular brown fat weight per 100g of body weight (bottom panel) in adult SHR control males (SHR ctl, black bars), SHR males programmed with maternal HSD (SHR prog, light-gray bars), SHR-*Zbtb16* control males (SHR-*Zbtb16* ctl, green bars) and SHR-*Zbtb16* males programmed with maternal HSD (SHR-*Zbtb16* prog, empty green bars). Within the graph, the significance levels of pair-wise comparisons by *post-hoc* Fisher’s least significant difference test of the two-way ANOVA with STRAIN and MATERNAL DIET as major factors are indicated as follows: **p < 0.01, ***p < 0.001.

We observed no difference in newborn offspring in terms of birth weight, sex ratio, or litter size between the dietary regimens or strains. Morphometric analysis of 6-month-old male offspring showed comparable body weights, relative weights of heart, liver, and adrenals among groups. Control SHR offspring (SHR ctl) had higher relative weights of retroperitoneal and epididymal fat pads compared to control SHR-*Zbtb16* (SHR-*Zbtb16* ctl; Table 1). Maternal HSD programming substantially increased brown fat weight (by 46.5% in SHR prog vs. SHR ctl, by 70.0% in SHR-*Zbtb16* prog vs. SHR-*Zbtb16* ctl) in offspring of both strains (Figure 8) and, only in SHR prog, we observed a 5.2% decrease in kidney weight.

**TABLE 1:** Morphometric profiles of adult SHR and SHR-*Zbtb16* male rats.

g or mg/100g body weight	SHR ctl	SHR prog	SHR-*Zbtb16* ctl	SHR-*Zbtb16* prog	STRAIN	MATERNAL DIET	STRAIN*MATERNAL DIET
Body weight (g)	312 ± 7	305 ± 6	298 ± 3	298 ± 5	0.19	0.61	0.61
Heart	0.38 ± 0.01	0.39 ± 0.01	0.39 ± 0.01	0.39 ± 0.01	0.62	0.69	0.62
Liver	2.85 ± 0.04	2.76 ± 0.04	2.74 ± 0.02	2.73 ± 0.03	0.17	0.34	0.42
Kidneys	0.68 ± 0.01	0.64 ± 0.01**^S^**	0.66 ± 0.01	0.64 ± 0.01	0.34	4.45.10^–4^	0.61
Adrenals (mg)	13.6 ± 0.6	14.3 ± 0.3	13.7 ± 0.2	13.1 ± 0.4	0.37	0.95	0.22
Brown fat (mg)	65.5 ± 3.7	96 ± 4.7**^S^**	53.5 ± 3.6	90.9 ± 3.8**^Z^**	0.15	2.94.10^–9^	0.61
RFP	1.02 ± 0.05	0.98 ± 0.06	0.78 ± 0.03**	0.93 ± 0.07	0.023	0.42	0.19
EFP	0.88 ± 0.03	0.89 ± 0.02	0.79 ± 0.01*	0.84 ± 0.04	0.028	0.42	0.61

Morphometric profiles of adult SHR and SHR-Zbtb16 male rats. Values are shown as mean ± SEM (g or mg, where indicated) for organ weights per 100 g of body weight. The last three columns show significance levels of STRAIN, MATERNAL DIET major factors, and their interaction within the two-way ANOVA. RFP, retroperitoneal fat pad; EFP, epididymal fat pad. The significance levels of pair-wise comparisons by post-hoc Fisher’s least significant difference test are indicated as follows: *p < 0.05, **p < 0.01, for comparison of SHR and SHR-Zbtb16 on the same diet; **S** indicates the difference between control and programmed SHR (kidneys p = 0.003; brown fat p = 6.17.10^–^^5^); **Z** indicates the difference between control and programmed SHR-Zbtb16 (brown fat p = 1.42.10^–^^6^).

### Metabolic Profile of Adult Male Offspring

**FIGURE 9:**
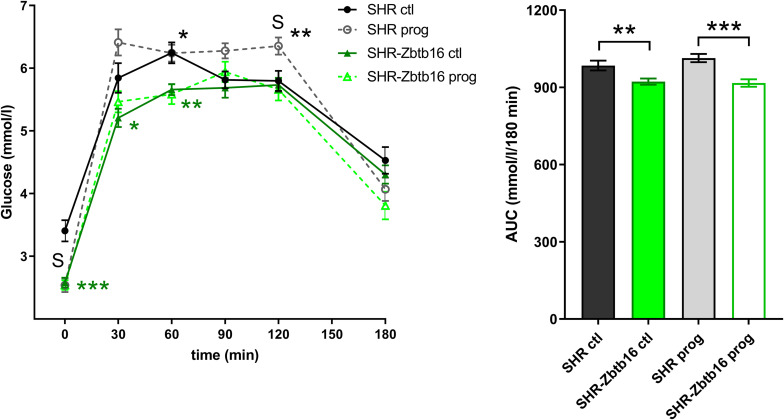
The oral glucose tolerance test (OGTT). The course of glycemic curves in adult SHR control males (SHR ctl, black circles), SHR males programmed with maternal HSD (SHR prog, empty gray circles), SHR-*Zbtb16* control males (SHR-*Zbtb16* ctl, dark green triangles), SHR-*Zbtb16* males programmed with maternal HSD (SHR-*Zbtb16* prog, light-green empty triangles) during the oral glucose tolerance test with corresponding areas under the curves (AUC). Data are expressed as mean ± SEM. Within the graph, the significance levels of pair-wise comparisons by repeated-measures two-way ANOVA (OGTT) and *post-hoc* Fisher’s test of the two-way ANOVA (AUC) ANOVA with STRAIN and MATERNAL DIET as major factors are indicated as follows: *p < 0.05, **p < 0.01, ***p < 0.001. Green asterisks (*) show strain differences between SHR ctl and SHR-*Zbtb16* ctl, black asterisks (*) show strain differences between SHR prog and SHR-*Zbtb16* prog rats. **S** represents differences between SHR ctl and SHR prog rats: *t* = 0 min^∗∗∗^, *t* = 120 min^∗^.

**FIGURE 10:**
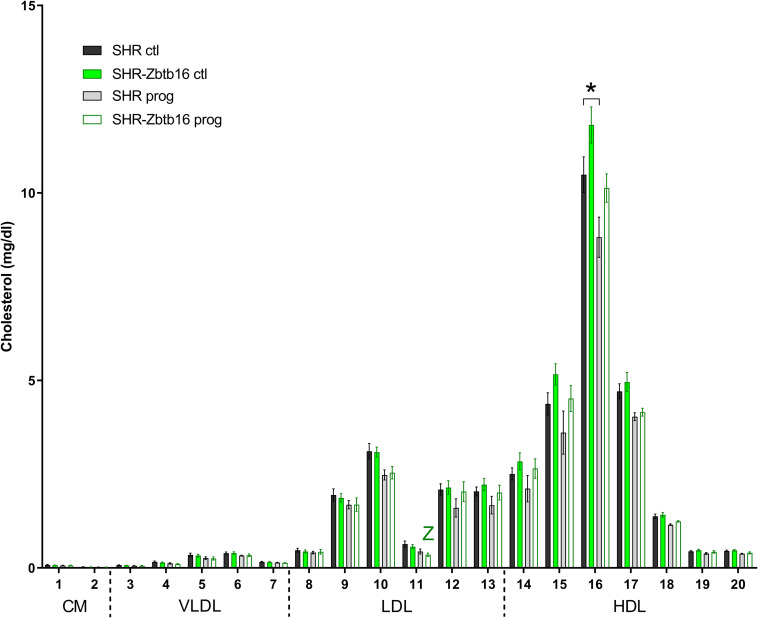
Cholesterol lipoprotein profile. The cholesterol content in 20 lipoprotein subfractions in adult SHR control males (SHR ctl, black bars), SHR males programmed with maternal HSD (SHR prog, light gray bars), SHR-*Zbtb16* control males (SHR-*Zbtb16* ctl, green bars) and SHR-*Zbtb16* males programmed with maternal HSD (SHR-*Zbtb16* prog, empty green bars). The significance levels of pair-wise comparisons by *post-hoc* Fisher’s least significant difference test of the two-way ANOVA with STRAIN and MATERNAL DIET as major factors are indicated as follows: *p < 0.05, **p < 0.01. **Z** represents differences in SHR-*Zbtb16*: Ch11^∗∗^ between control and programmed males. The allocation of individual lipoprotein subfractions to major lipoprotein classes is shown in order of particle’s decreasing size from left to right. CM, chylomicron; VLDL, very low-density lipoprotein; LDL, low density lipoprotein; HDL, high density lipoprotein.

SHR ctl showed significantly higher fasting insulin concentration compared to SHR-*Zbtb16* ctl rats (Figure 8). During the oral glucose tolerance test, SHR-*Zbtb16* ctl and SHR-*Zbtb16* prog showed better glucose tolerance in comparison with SHR ctl and SHR prog, respectively. SHR prog males had significantly lower fasting glycemia and, at the same time, significantly higher glycemia 2 h after the glucose load (Figure 9) compared to SHR ctl. Offspring of both strains showed similar profiles of cholesterol and triacylglycerols distribution into lipoprotein fractions. There was virtually no programming effect of maternal HSD on cholesterol levels in offspring of both strains except a modest decrease of cholesterol in single LDL fraction in SHR-*Zbtb16* prog (Figure 10). However, in both SHR prog and SHR-*Zbtb16* prog, we observed a reduction in triacylglycerols content of small and very small LDL particles compared to SHR ctl and SHR-*Zbtb16* ctl, respectively (Table 2). Also, SHR-*Zbtb16* prog showed a decrease of triacylglycerols in medium HDL compared to SHR-*Zbtb16* ctl.

**TABLE 2:** Triacylglycerol lipoprotein profile of control and programmed male offspring.

Class	Fraction	SHR ctl (mg/dl)	SHR prog (mg/dl)	SHR-*Zbtb16* ctl (mg/dl)	SHR-*Zbtb16* prog (mg/dl)
CM (>80 nm)	01	0.60 ± 0.09	0.46 ± 0.08	0.58 ± 0.05	0.53 ± 0.13
	02	0.26 ± 0.04	0.19 ± 0.03	0.24 ± 0.02	0.21 ± 0.04
VLDL (30–80 nm)	03	0.66 ± 0.11	0.45 ± 0.08	0.59 ± 0.08	0.46 ± 0.08
	04	1.78 ± 0.41	1.14 ± 0.27	1.48 ± 0.26	0.99 ± 0.21
	05	3.85 ± 0.76	2.46 ± 0.48	3.61 ± 0.48	2.55 ± 0.53
	06	2.73 ± 0.37	1.99 ± 0.18	2.91 ± 0.24	2.35 ± 0.35
	07	0.84 ± 0.09	0.67 ± 0.04	0.88 ± 0.06	0.74 ± 0.08
LDL (16–30 nm)	08	1.26 ± 0.13	1.01 ± 0.04	1.31 ± 0.10	1.12 ± 0.05
	09	3.28 ± 0.21	2.66 ± 0.13	3.53 ± 0.19	2.94 ± 0.09**^Z^**
	10	3.62 ± 0.22	2.77 ± 0.09**^S^**	3.82 ± 0.19	2.99 ± 0.11**^Z^**
	11	1.32 ± 0.10	0.99 ± 0.06**^S^**	1.36 ± 0.07	1.05 ± 0.05**^Z^**
	12	0.39 ± 0.04	0.27 ± 0.01**^S^**	0.39 ± 0.02	0.31 ± 0.01**^Z^**
	13	0.26 ± 0.02	0.19 ± 0.0 1**^S^**	0.25 ± 0.01	0.20 ± 0.01**^Z^**
HDL (8–16 nm)	14	0.14 ± 0.01	0.11 ± 0.01	0.15 ± 0.01	0.12 ± 0.01
	15	0.16 ± 0.01	0.13 ± 0.02	0.18 ± 0.01	0.15 ± 0.01
	16	0.32 ± 0.03	0.27 ± 0.01	0.34 ± 0.02	0.29 ± 0.02
	17	0.12 ± 0.01	0.10 ± 0.01	0.13 ± 0.01	0.10 ± 0.01**^Z^**
	18	0.35 ± 0.02	0.33 ± 0.03	0.40 ± 0.04	0.395 ± 0.01
	19	0.21 ± 0.03	0.22 ± 0.05	0.29 ± 0.03	0.26 ± 0.08
	20	0.83 ± 0.03	0.71 ± 0.02	0.81 ± 0.05	0.71 ± 0.03

The triacylglycerol content in 20 lipoprotein subfractions in adult SHR control males (SHR ctl), SHR males programmed with maternal HSD (SHR prog), SHR-Zbtb16 control males (SHR-Zbtb16 ctl) and SHR-Zbtb16 males programmed with maternal HSD (SHR-Zbtb16 prog). The allocation of individual lipoprotein subfractions to major lipoprotein classes is shown in order of particle’s decreasing size from top to bottom. CM-chylomicron, VLDL-very low density lipoprotein, LDL-low density lipoprotein, HDL-high density lipoprotein. The significance levels of group comparison by post-hoc Fisher’s least significant difference test of the two-way ANOVA with STRAIN and MATERNAL DIET as major factors are indicated as follows: *p < 0.05, **p < 0.01, ***p < 0.001. **S** represents differences between SHR control and SHR programmed males in fractions 10**, 11*, 12**, and 13*. **Z** represents differences between SHR-Zbtb16 control and SHR-Zbtb16 programmed males in fractions 9*, 10***, 11**, 12*, 13*, and 17*.

### Transcriptomic Profiles of Adult Male Offspring

**FIGURE 11:**
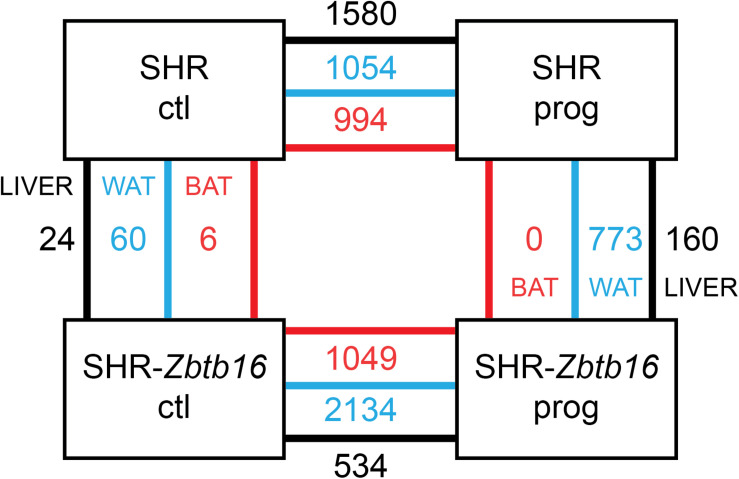
Schematic depiction of transcriptome results comparison in adult male offspring of SHR and SHR-*Zbtb16* rat strains. Numbers of significantly differentially expressed transcripts (FDR <0.05, >1.5-fold-change) between control (ctl) and maternally programmed (prog) groups of both strains are shown for liver (black lines), white adipose tissue (WAT, blue lines) and brown adipose tissue (BAT, red lines).

**FIGURE 12:**
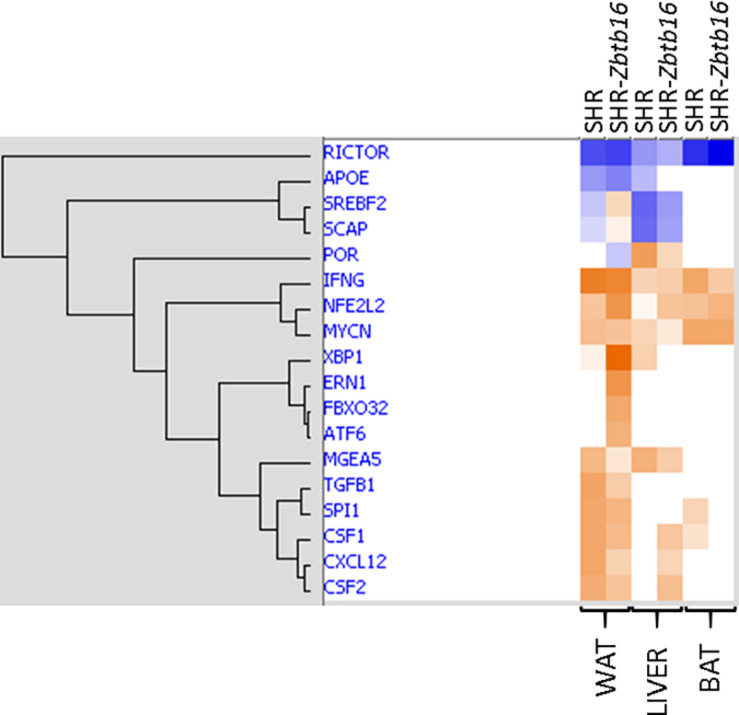
Comparison of maternal HSD programming effect on activation (shades of orange) or inhibition (shades of blue) of upstream regulators in white adipose tissue (WAT), liver and brown adipose tissue (BAT) in male offspring of HSD- vs. standard diet-fed rat dams of SHR and SHR-*Zbtb16* rat strains. Hierarchical clustering and calculation of activation z-scores were performed using Ingenuity Pathways Analysis.

**FIGURE 13:**
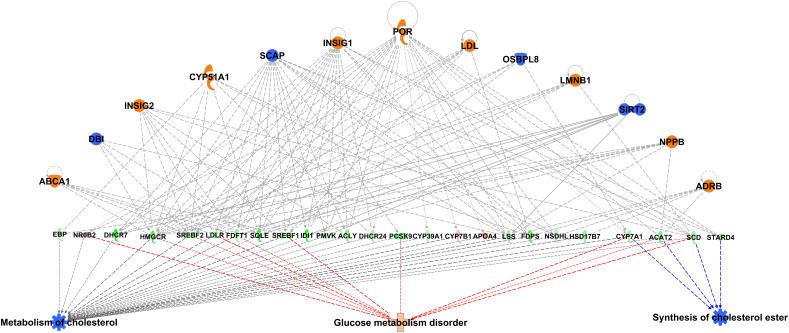
Mechanistic network summarizing main effects of maternal HSD programming effect on activation (shades of orange) or inhibition (shades of blue) of upstream regulators in white adipose tissue, liver, and brown adipose tissue in male offspring of HSD- vs. standard diet-fed rat dams of SHR and SHR-*Zbtb16* rat strains. The programming effect on the expression of genes significantly differentially expressed in the above datasets is shown in shades of green (downregulation) or red (upregulation). Derivation of the network was performed using Ingenuity Pathways Analysis.

As summarized in Figure 11, SHR ctl and SHR-*Zbtb16* ctl differed in expression of a limited number of transcripts, and there was no common transcript among the three tissues. However, when comparing SHR prog vs. SHR-*Zbtb16* prog, we observed several hundred differentially expressed transcripts in white adipose tissue and liver. In contrast, none of the transcripts reached the significance threshold for differential expression within the transcriptomic profile of brown adipose tissue between the two strains. Summary of all differentially expressed transcripts including selected transcripts validated by qPCR is provided in Supplementary Material. Among the genes with the most significant difference in expression between SHR prog and SHR-*Zbtb16* prog white adipose tissue were angiopoietin-like 8 (*Angptl8*, downregulated in SHR-*Zbtb16*) and lipase I (*LipI*, upregulated in SHR-*Zbtb16*). In contrast, the most upregulated gene in the liver of SHR-*Zbtb16* prog was cytochrome P450 family 7 subfamily A member 1 (*Cyp7a1*) and among the downregulated genes we found a cluster of olfactory receptors. The effect of programming by maternal HSD feeding on change of transcriptomic profile was evident in both strains and all analyzed tissues (Figure 11). Interestingly, while there was a limited overlap among the transcripts induced or repressed by maternal HSD between the two strains, the analysis of canonical pathways, upstream regulators and mechanistic networks revealed mostly comparable results. Figure 12 shows the result of the comparison of HSD programming effect for upstream regulators in the two strains across all tissues. Except for the slightly distinct pattern of white adipose tissue in SHR-*Zbtb16*, the profile is very similar in the respective tissues of both strains. In an attempt to summarize the mechanism underlying observed metabolic shifts on the transcriptomic level, we have derived a mechanistic network pinpointing the key nodes with the highest score connecting the differentially expressed transcripts, their upstream regulators and downstream (patho)physiological processes (Figure 13). The expression changes of 25 transcripts converged to three major metabolic processes perturbed by HSD-induced programming, namely metabolism of cholesterol, glucose, and synthesis of cholesterol esters. While this particular pattern was most evident in SHR rats, we observed a similar pattern in SHR-*Zbtb16*.

## Discussion

In the current paper, we show that the administration of HSD to pregnant rat dams significantly affects not only their metabolic profiles but, to a lesser extent, also the metabolic and transcriptomic profiles of their inbred offspring. The effects of modulation of maternal macronutrient consumption have been extensively reviewed recently (Kereliuk et al., 2017; Nicholas and Ozanne, 2019). We report that maternal HSD administration substantially increased brown fat weight in adult male offspring of both strains. It points to an interesting connotation with a study showing that brown fat is a critical regulator of the effect of maternal nutritional programming (Dumortier et al., 2017). We recently observed a similar effect in the second generation of both SHR and SHR-*Zbtb16* offspring of HSD-fed rat dams (Skolnikova et al., 2020). However, the transcriptomic profile in both strains suggests a compromised function of brown adipose tissue. The most enriched canonical pathway in both strains was Mitochondrial dysfunction, and we observed and validated a 2-fold increase in expression of hydroxysteroid 11-beta dehydrogenase 1 (*Hsd11b1*), overexpression of which was shown to suppress brown adipocyte function (Liu et al., 2013). This is complemented by a significant decrease of Iodothyronine deiodinase 2 (*Dio2*) gene expression, a major activating deiodinase (Gereben et al., 2015). Most pronounced programming effect across all tissues at the level of individual upstream regulators was inhibition of RICTOR, a regulatory subunit of the mammalian target of rapamycin complex 2. Loss of RICTOR leads to the global dampening of insulin/AKT signaling (Entwisle et al., 2020). Although this observation did not translate to change of global glucose tolerance in the programmed rat offspring, subtler change in insulin resistance of peripheral tissues cannot be excluded, as suggested by the 3-fold (2-fold in SHR-*Zbtb16*) reduction of Glut4 expression in white adipose tissue of SHR male offspring and, in a network perspective, the glucose metabolism disorder activated node. However, it seems that this effect may be secondary to changes in the expression of lipid metabolism-related transcripts, including *Srebf1*, *Srebf2*, *Pcsk9*, *Scd*, *Acat2*, and others, observed particularly in livers of both strains. A systematic shift in expression of more than 20 transcripts indicated a substantial downregulation of cholesterol metabolism and synthesis of cholesterol esters. A related result contrasting several previous studies (Sedova et al., 2007; D’Alessandro et al., 2014; Kereliuk et al., 2017) was the slight improvement of the lipid profile of programmed offspring, particularly in the class of LDL-TG, associated with low-grade systemic inflammation and coronary artery disease in humans (Silbernagel et al., 2019). Providing pregnant female rats with high-sucrose diet *ad libitum* resulted in higher fasting glycemia and elevation of serum cholesterol and triacylglycerols in chylomicrons and LDL particles in rat dams of both strains. This is in line with observations of previous studies focused on the consumption of diets high in sugar (Kendig et al., 2015) even after just 10 days, when the metabolic screening was performed. The consumption of HSD was elevated in pregnancy in both strains, most likely due to the high palatability of the diet and sweet taste preference of rats (Schulte et al., 2015). There was, however, no differential impact on body weight between strains or diet groups during pregnancy until the second week of lactation, when the HSD-fed groups became significantly lighter than STD-fed groups. While most of the so far published studies on effects on maternal programming do not follow maternal weight postpartum, let alone under different dietary conditions, our finding is in agreement with a previously published account on sucrose-fed rat dams (Kendig et al., 2015). In general, rodent fat deposition is increasing during pregnancy, and with lactation, the storage of lipids in adipose tissue is lowering as lipids are being transferred into milk (Stuebe and Rich-Edwards, 2009). The significant reduction of body weight in lactating females fed HSD may be related to altered carbohydrate source in the diet interacting with demands of lactation as a similar drop was observed in rat dams fed a low-protein diet (Pine et al., 1994). Indeed, the HSD administration can be envisioned as an enhanced “stress” or metabolic challenge, resulting in worse coping in the early postpartum period (Aiken et al., 2019). On the other hand, we did not observe any effect on the growth rates between the offspring of HSD and STD-fed SHR and SHR-*Zbtb16* dams. Most importantly, we show that the effect of maternal nutritional programming is dependent on the genomic background it acts upon. The variant *Zbtb16* allele present in the SHR-*Zbtb16* strain is likely responsible for several subtle distinct effects of maternal HSD on adult male offspring, including less pronounced response of insulin levels and particularly the transcriptome shifts, most apparent in white adipose tissue. ZBTB16 is a downstream effector for PGC-1-controlled gluconeogenesis, and at the same time, ZBTB16 negatively regulates the insulin signaling pathway by decreasing the phosphorylation of IRS1, Akt, and FoxO1 in normal mice. Liver-specific knockdown of *Zbtb16* relieved hyperglycemia in db/db mice and led to decreased insulin levels, improved glucose and pyruvate tolerance, and insulin sensitivity (Chen et al., 2014). We showed earlier that SHR*-Lx* congenic strain carrying the same 2 kb-deletion in an intron of *Zbtb16* as the SHR-*Zbtb16* strain displays higher sensitivity to dexamethasone-induced insulin resistance of the skeletal muscle when compared to SHR controls (Seda et al., 2005) and this effect was persistent in SHR-*Zbtb16* itself (Krupkova et al., 2018). The limitations of the current study include the use of only male offspring as sex-specific metabolic syndrome phenotype was previously demonstrated (Seda et al., 2008) and programming of several related traits is sex-dependent (Litzenburger et al., 2020). We focused the current study on effects of a minute genetic difference, therefore we opted to use only males of a highly inbred model of metabolic syndrome to maximize the homogeneity of our control and experimental groups and avoid e.g., the potential effects of estrous cycle on gene expression etc. In a smaller-scale study involving female offspring of both SHR and SHR-*Zbtb16* strains, we found that maternal HSD administration led to an increase in fasting insulinemia in both strains and, exclusively in SHR females, to an improvement of glucose tolerance (Skolnikova et al., 2020). By assessing the effects of maternal programming in genetically distinct models, it may become possible to elucidate the genetic component of susceptibility to dietary regimens in the early development (Sedova et al., 2007). Also, as the experimental protocol was set up so that HSD was administered throughout the pregnancy and lactation, it is impossible to distinguish which of the potentially critical periods (pre-conception, gestation, lactation) is more influential concerning the observed phenotypic effects. Further studies should address in detail the mechanisms and pathways, through which the *Zbtb16* mediates the distinct programming effect since its expression on the level of mRNA was not changed in our study. Also, without confirmation on a mechanistic level, the transcriptome-derived relationships are only indicative of possible underlying processes that still need to be validated. In summary, the presented results show that HSD administration to pregnant rats leads to increase in brown adipose tissue weight and slight reduction of LDL-TG in their adult male offspring. At the same time, maternal HSD administration triggered substantial, strain-specific shifts in transcriptomes of liver, white and brown adipose tissues. The variant *Zbtb16* allele led to strain-specific effect of HSD-induced changes in transcriptomic profiles of the offspring with a limited effect on induced metabolic changes.

## Data Availability Statement

The datasets generated for this study can be found in the ArrayExpress repository, Experiment ArrayExpress accession: E-MTAB-6838 (https://www.ebi.ac.uk/arrayexpress/experiments/E-MTAB-6838).

## Ethics Statement

The animal study was reviewed and approved by the Ethical Committee of the First Faculty of Medicine of the Charles University and the Ministry of Education, Youth and Sports (protocol no. MSMT-14076/2015-14).

## Author Contributions

EŠ, LŠ, BC, and AK carried out the experimental components of the study. EŠ and OŠ drafted the manuscript. FL participated in the design of the study and performed the statistical analysis. OŠ, EŠ, and LŠ conceived the study and participated in its design and coordination. All authors participated in the manuscript preparation, read and approved the final manuscript.

## Conflict of Interest

The authors declare that the research was conducted in the absence of any commercial or financial relationships that could be construed as a potential conflict of interest.

## Footnotes

1 https://www.ebi.ac.uk/arrayexpress/experiments/E-MTAB-6838
