# Supplementary material for: Maternal High-Sucrose Diet Affects Phenotype Outcome in Adult Male Offspring: Role of Zbtb16
Source: Front Genet. 2020 Sep 11;11:529421. doi: 10.3389/fgene.2020.529421 (PMC7518089; doi:10.3389/fgene.2020.529421)
Supplement: Supplementary file 1 [file Data_Sheet_1.docx]

**SUPPLEMENTARY MATERIAL**

**Maternal high-sucrose diet affects phenotype outcome in adult male offspring: role of *Zbtb16***

**Elena Školníková, Lucie Šedová, Blanka Chylíková, Adéla Kábelová, František Liška, Ondřej Šeda**

Institute of Biology and Medical Genetics, First Faculty of Medicine, Charles University and the General University Hospital, Prague, Czech Republic

Corresponding author: Ondřej Šeda

Institute of Biology and Medical Genetics

First Faculty of Medicine, Charles University,

Albertov 4, 12800 Prague 2, Czech Republic

Tel: +420 224968180; email: [oseda@lf1.cuni.cz](mailto:oseda@lf1.cuni.cz)

**Supplementary Table 1: Diet composition**

|  | **carbohydrates (kJ%)** | |  | **protein (kJ%)** | **fat (kJ%)** | **ME (MJ/kg)** |
| --- | --- | --- | --- | --- | --- | --- |
| **Standard diet** | 64 | starch | | 26 | 10 | 13.6 |
| **High sucrose diet** | 70 | sucrose | | 19.6 | 10.4 | 12.9 |

*Macronutrient composition of standard and high-sucrose diets. ME – estimated metabolizable energy, MJ – megajoule, kg – kilogram, kJ – kilojoule. The amount of micronutrients and vitamins is identical in the two diets.*

| Cholesterol  Class | mg/dl | SHR ctl  adult | SHR ctl  pregnant | SHR HSD  pregnant | SHR-*Zbtb16*  ctl adult | SHR-*Zbtb16*  ctl pregnant | SHR-*Zbtb16*  HSD |
| --- | --- | --- | --- | --- | --- | --- | --- |
| Chylomicrons | C1 | 0.07±0.01 | 0.10±0.02 | 0.31±0.08 A***/P*** | 0.06±0.01 | 0.09±0.01 | 0.31±0.05 A***/P*** |
|  | C2 | 0.03±0.00 | 0.05±0.01 | 0.13±0.03 A***/P** | 0.02±0.01 | 0.04±0.01 | 0.13±0.02 A***/P*** |
| Very low-density lipoproteins | C3 | 0.07±0.01 | 0.11±0.01 | 0.26±0.05 A***/P*** | 0.06±0.01 | 0.11±0.01 | 0.28±0.04 A***/P*** |
|  | C4 | 0.13±0.02 | 0.19±0.03 | 0.39±0.07 A***/P** | 0.12±0.02 | 0.21±0.03 | 0.43±0.07 A***/P** |
|  | C5 | 0.19±0.02 | 0.29±0.03 | 0.45±0.06 A***/P* | 0.18±0.03 | 0.33±0.05 A* | 0.53±0.07 A***/P** |
|  | C6 | 0.24±0.02 | 0.40±0.03 A*** | 0.39±0.03 A**/P** | 0.20±0.02 | 0.38±0.04 A*** | 0.50±0.04 A***/P** |
|  | C7 | 0.10±0.01 | 0.17±0.02 A** | 0.12±0.00 A* | 0.07±0.01 | 0.14±0.02 A** | 0.17±0.02 A***/# |
| Low-density lipoproteins | C8 | 0.32±0.04 | 0.62±0.08 A*** | 0.47±0.05 | 0.29±0.02 | 0.58±0.05 A** | 0.67±0.08 A***/# |
|  | C9 | 1.00±0.11 | 1.36±0.18 | 0.80±0.07 | 0.90±0.04 | 1.26±0.15 | 1.04±0.18 |
|  | C10 | 1.73±0.09 | 2.91±0.31 A*** | 1.68±0.13 P*** | 1.76±0.11 | 2.46±0.24 A* | 2.05±0.20 |
|  | C11 | 0.14±0.05 | 0.43±0.12 A** | 0.09±0.02 P** | 0.18±0.07 | 0.27±0.07 | 0.11±0.03 |
|  | C12 | 1.90±0.23 | 4.28±0.35 A*** | 4.10±0.53 A** | 2.26±0.40 | 4.42±0.69 A** | 5.53±0.34 A***/# |
|  | C13 | 2.11±0.22 | 3.54±0.25 A** | 3.40±0.38 A* | 2.47±0.37 | 3.86±0.53 A** | 4.39±0.24 A*** |
| High-density lipoproteins | C14 | 3.24±0.36 | 4.71±0.43 A* | 4.80±0.49 A* | 3.60±0.60 | 4.84±0.66 | 5.51±0.32 A* |
|  | C15 | 6.55±0.71 | 8.19±0.90 | 8.18±0.47 | 6.88±0.78 | 8.32±0.88 | 8.69±0.48 |
|  | C16 | 15.80±0.80 | 18.09±1.24 | 19.43±0.75 A* | 16.06±1.33 | 18.29±1.17 | 18.16±0.55 |
|  | C17 | 7.12±0.21 | 7.44±0.28 | 7.92±0.31 | 7.02±0.35 | 7.91±0.27 | 7.48±0.45 |
|  | C18 | 2.31±0.04 | 2.59±0.16 | 2.83±0.10 A** | 2.64±0.09 # | 2.91±0.10 # | 2.77±0.12 |
|  | C19 | 0.77±0.03 | 0.82±0.08 | 0.76±0.05 | 0.72±0.04 | 0.85±0.03 | 0.82±0.05 |
|  | C20 | 0.54±0.02 | 0.69±0.04 A*** | 0.69±0.02 A*** | 0.56±0.03 | 0.66±0.03 A* | 0.63±0.02 |

**Supplementary Table 2.** The cholesterol content in 20 lipoprotein subfractions in adult control (ctl) SHR females, pregnant control SHR females, pregnant SHR females fed HSD, adult control SHR-*Zbtb16* females, pregnant control SHR-Zbtb16 females and pregnant SHR-*Zbtb16* females fed HSD. The allocation of individual lipoprotein subfractions to major lipoprotein classes is shown in order of particle’s decreasing size from top to bottom. The significance levels of group comparison by post hoc Fisher’s least significant difference test of two-way ANOVA with STRAIN and DIET as major factors are indicated as follows: * p<0.05, ** p<0.01, *** p<0.001. A represents differences between the pregnant control or pregnant HSD group and their adult control group, P represents differences between the pregnant control and pregnant HSD group in the same strain. Strain differences are represented by hashtags (#) and they correspond to the pairwise groups (adult SHR control – adult SHR-*Zbtb16* control).

| Triacylglycerols  Class | mg/dl | SHR ctl  adult | SHR ctl  pregnant | SHR HSD  pregnant | SHR-*Zbtb16*  ctl adult | SHR-*Zbtb16*  ctl pregnant | SHR-*Zbtb16*  HSD |
| --- | --- | --- | --- | --- | --- | --- | --- |
| Chylomicrons | TG1 | 0.24±0.06 | 0.55±0.22 | 4.50±1.62 A***/P** | 0.21±0.08 | 0.46±0.13 | 3.84±0.92 A**/P** |
|  | TG2 | 0.16±0.04 | 0.33±0.11 | 1.85±0.59 A***/P** | 0.15±0.04 | 0.37±0.08 | 1.75±0.42 A***/P** |
| Very low-density lipoproteins | TG3 | 0.50±0.10 | 0.98±0.27 | 4.01±1.16 A***/P** | 0.56±0.14 | 1.26±0.29 | 3.87±0.94 A***/P** |
|  | TG4 | 1.00±0.16 | 1.79±0.46 | 5.32±1.47 A**/P** | 1.29±0.32 | 2.69±0.68 | 5.41±1.25 A**/P* |
|  | TG5 | 1.30±0.13 | 2.11±0.52 | 4.45±1.17 A**/P* | 1.75±0.37 | 3.17±0.78 | 4.87±1.00 A** |
|  | TG6 | 0.83±0.07 | 1.47±0.24 | 1.89±0.38 A* | 1.01±0.15 | 1.74±0.30 | 2.22±0.37 A** |
|  | TG7 | 0.34±0.03 | 0.69±0.09 A** | 0.55±0.07 | 0.37±0.04 | 0.72±0.11 A** | 0.71±0.09 A** |
| Low-density lipoproteins | TG8 | 0.51±0.05 | 1.17±0.15 A*** | 0.79±0.07 | 0.56±0.06 | 1.22±0.17 A*** | 1.11±0.13 A** |
|  | TG9 | 1.83±0.11 | 2.84±0.24 A** | 1.67±0.11 P*** | 1.91±0.16 | 2.85±0.27 A** | 2.12±0.26 P* |
|  | TG10 | 2.35±0.03 | 3.42±0.39 A** | 1.47±0.14 A*/P*** | 2.59±0.17 | 3.02±0.32 | 1.73±0.20 A*/P*** |
|  | TG11 | 0.85±0.02 | 1.37±0.19 A** | 0.49±0.06 A*/P*** | 0.95±0.06 | 1.09±0.14 | 0.55±0.05 A*/P** |
|  | TG12 | 0.20±0.01 | 0.51±0.07 A*** | 0.23±0.04 P*** | 0.28±0.02 | 0.47±0.07 A** | 0.34±0.04 |
|  | TG13 | 0.15±0.01 | 0.32±0.04 A*** | 0.16±0.03 P*** | 0.17±0.01 | 0.26±0.04 A* | 0.20±0.02 |
| High-density lipoproteins | TG14 | 0.08±0.01 | 0.19±0.02 A*** | 0.14±0.02 A* | 0.09±0.01 | 0.17±0.02 A** | 0.18±0.02 A** |
|  | TG15 | 0.13±0.02 | 0.22±0.04 | 0.19±0.04 | 0.14±0.02 | 0.21±0.04 | 0.24±0.04 A* |
|  | TG16 | 0.31±0.02 | 0.47±0.06 | 0.46±0.08 | 0.32±0.04 | 0.44±0.06 | 0.50±0.07 A* |
|  | TG17 | 0.08±0.01 | 0.08±0.03 | 0.12±0.03 | 0.08±0.02 | 0.11±0.02 | 0.16±0.03 A* |
|  | TG18 | 0.31±0.04 | 0.53±0.11 A* | 0.52±0.09 | 0.33±0.07 | 0.42±0.05 | 0.52±0.04 |
|  | TG19 | 0.25±0.06 | 0.51±0.11 A* | 0.47±0.13 | 0.24±0.07 | 0.35±0.03 | 0.40±0.05 |
|  | TG20 | 0.61±0.01 | 0.77±0.07 A* | 0.76±0.04 | 0.65±0.05 | 0.77±0.07 | 0.81±0.05 A* |

**Supplementary Table 3.** The triacylglycerol content in 20 lipoprotein subfractions in adult control SHR females, pregnant control SHR females, pregnant SHR females fed HSD, adult control SHR-*Zbtb16* females, pregnant control SHR-*Zbtb16* females and pregnant SHR-*Zbtb16* females fed HSD. The allocation of individual lipoprotein subfractions to major lipoprotein classes is shown in order of particle’s decreasing size from top to bottom. The significance levels of group comparison by post hoc Fisher’s least significant difference test of two-way ANOVA with STRAIN and DIET as major factors are indicated as follows: * p<0.05, ** p<0.01, *** p<0.001. A represents differences between the pregnant control or pregnant HSD group and their adult control group, P represents differences between the pregnant control and pregnant HSD group in the same strain.

| Gene symbol | Gene name | P (SHR prog vs. SHR ctl) | Fold change | P (SHR-*Zbtb16* prog vs. SHR-*Zbtb16* ctl) | Fold change |
| --- | --- | --- | --- | --- | --- |
| **Brown adipose tissue** | | | | | |
| *Dio2* | Iodothyronine deiodinase 2 | 3.8E-05 | -2.8 | 4.1E-04 | -2.3 |
| *Sod1* | Superoxide dismutase 1 | 1.6E-07 | 1.6 | 2.4E-09 | 1.7 |
| *Cox8b* | Cytochrome c oxidase subunit VIIIb | 3.7E-10 | 1.9 | 1.5E-16 | 3.1 |
| *Hsd11b1* | Hydroxysteroid 11-beta dehydrogenase 1 | 2.2E-05 | 2.0 | n.s. | n.s. |
| **White adipose tissue** | | | | | |
| *Slc2a4* | Solute carrier family 2 member 4 | 4.9E-08 | -3.0 | 1.6E-04 | -1.9 |
| *Vegfb* | Vascular endothelial growth factor B | 3.7E-05 | -2.2 | 9.9E-06 | -2.3 |
| *Hsd11b1* | Hydroxysteroid 11-beta dehydrogenase 1 | 3.7E-06 | 2.2 | 7.0E-04 | 1.7 |
| *Apoe* | Apolipoprotein E | 6.7E-09 | 3.6 | 4.1E-08 | 3.2 |
| **Liver** | | | | | |
| *Pcsk9* | Proprotein Convertase Subtilisin/Kexin Type 9 | 3.5E-10 | -4.5 | 7.7E-08 | -3.3 |
| *Sqle* | Squalene epoxidase | 4.4E-10 | -9.5 | 5.3E-06 | -4.2 |
| *Srebf1* | Sterol Regulatory Element Binding Transcription Factor 1 | 1.1E-04 | -2.1 | 1.0E-04 | -2.1 |
| *Nr0b2* | Nuclear Receptor Subfamily 0 Group B Member 2 | 3.6E-04 | 2.6 | n.s. | n.s. |
| *Acot1* | Acyl-CoA Thioesterase 1 | 6.6E-14 | 16.3 | 5.0E-11 | 8.9 |

**Supplementary Table 4.** Offspring tissue-specific transcripts and their expression change in response to high-sucrose diet administration to rat dams

*Transcripts validated by qPCR and their expression changes in response to nutritional programming by high-sucrose diet administration to SHR and SHR-Zbtb16 rat dams.*


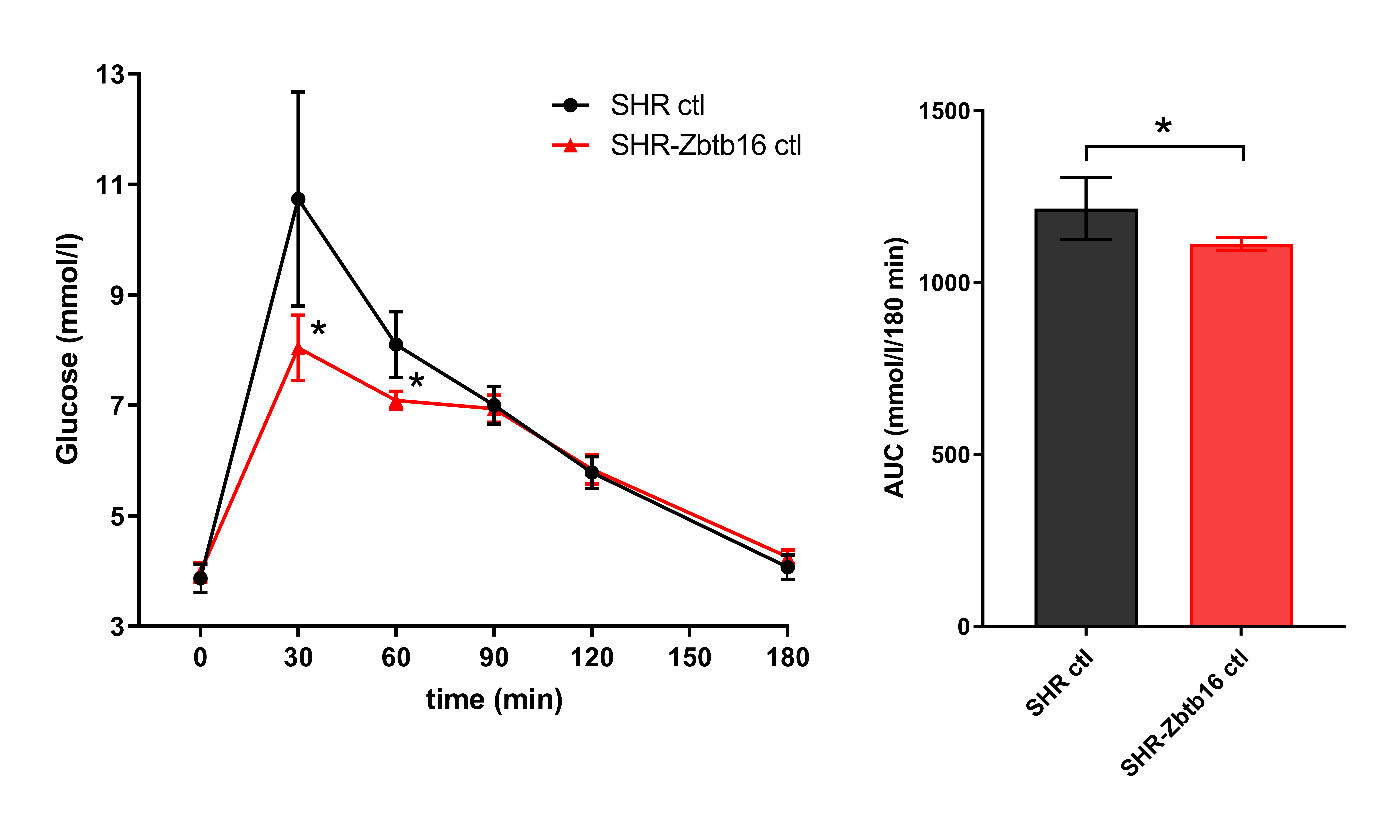


**Supplementary Figure 1.** The course of glycaemic curves in SHR females (black circles) and pregnant SHR-*Zbtb16* females (red triangles) before pregnancy during the oral glucose tolerance test OGTT with corresponding areas under the curves (AUC). Data are expressed as mean ± SEM. Within the graph, the significance levels of pairwise comparisons between strains by post-hoc Fisher's test of the two-way ANOVA with STRAIN and DIET as major factors are indicated as follows: * p<0.05.


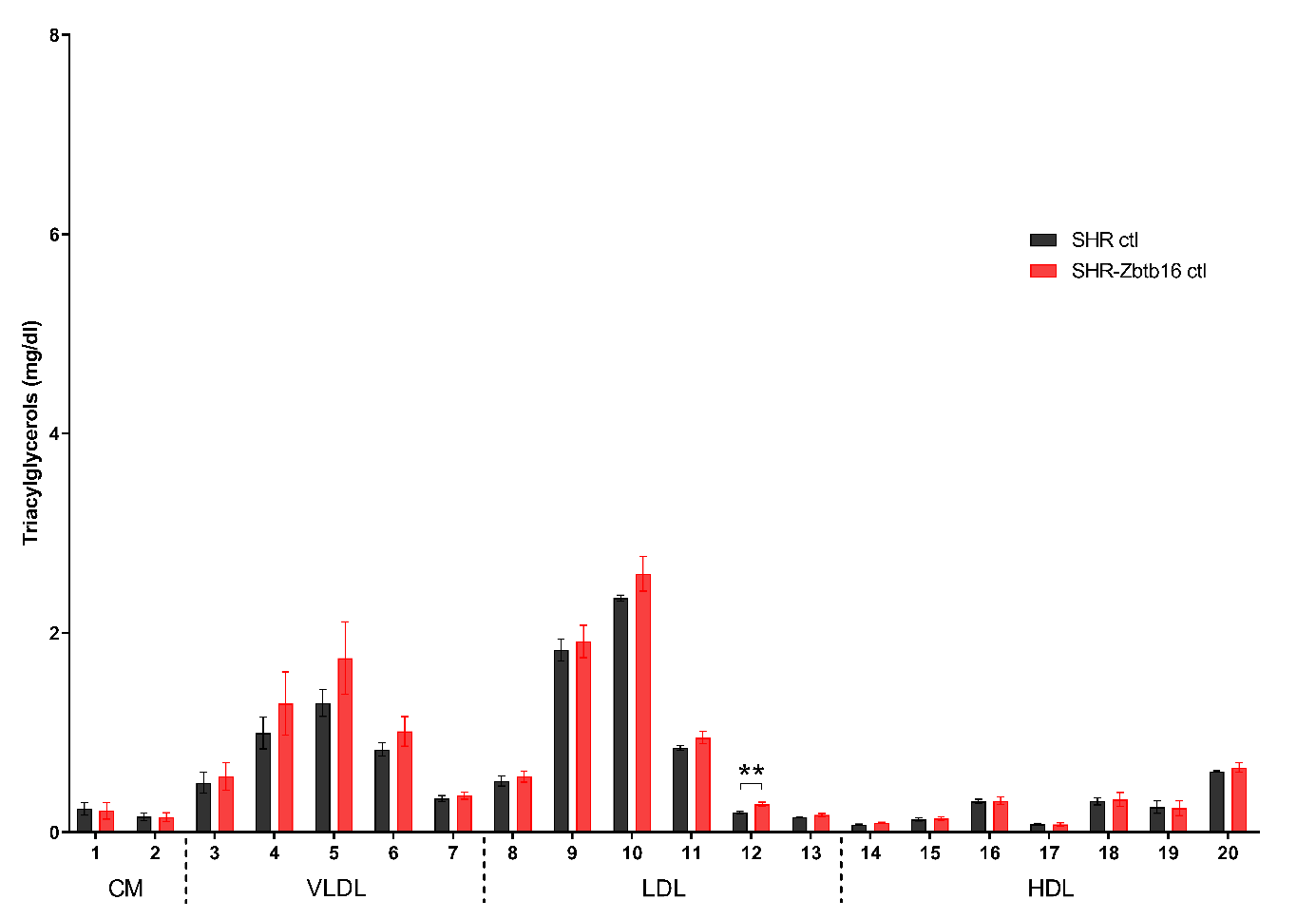


**Supplementary Figure 2.** The triacylglycerol content in 20 lipoprotein subfractions in SHR female rats (black bars) and SHR-*Zbtb16* female rats (red bars) before pregnancy. Within the graph, the significance levels of pairwise comparisons between strains by post-hoc Fisher's test of the two-way ANOVA with STRAIN and DIET as major factors are indicated as follows: ** p<0.01. The allocation of individual lipoprotein subfractions to major lipoprotein classes is shown in order of particle's decreasing size from left to right. CM-chylomicron, VLDL-very low-density lipoprotein, LDL-low density lipoprotein, HDL-high density lipoprotein.


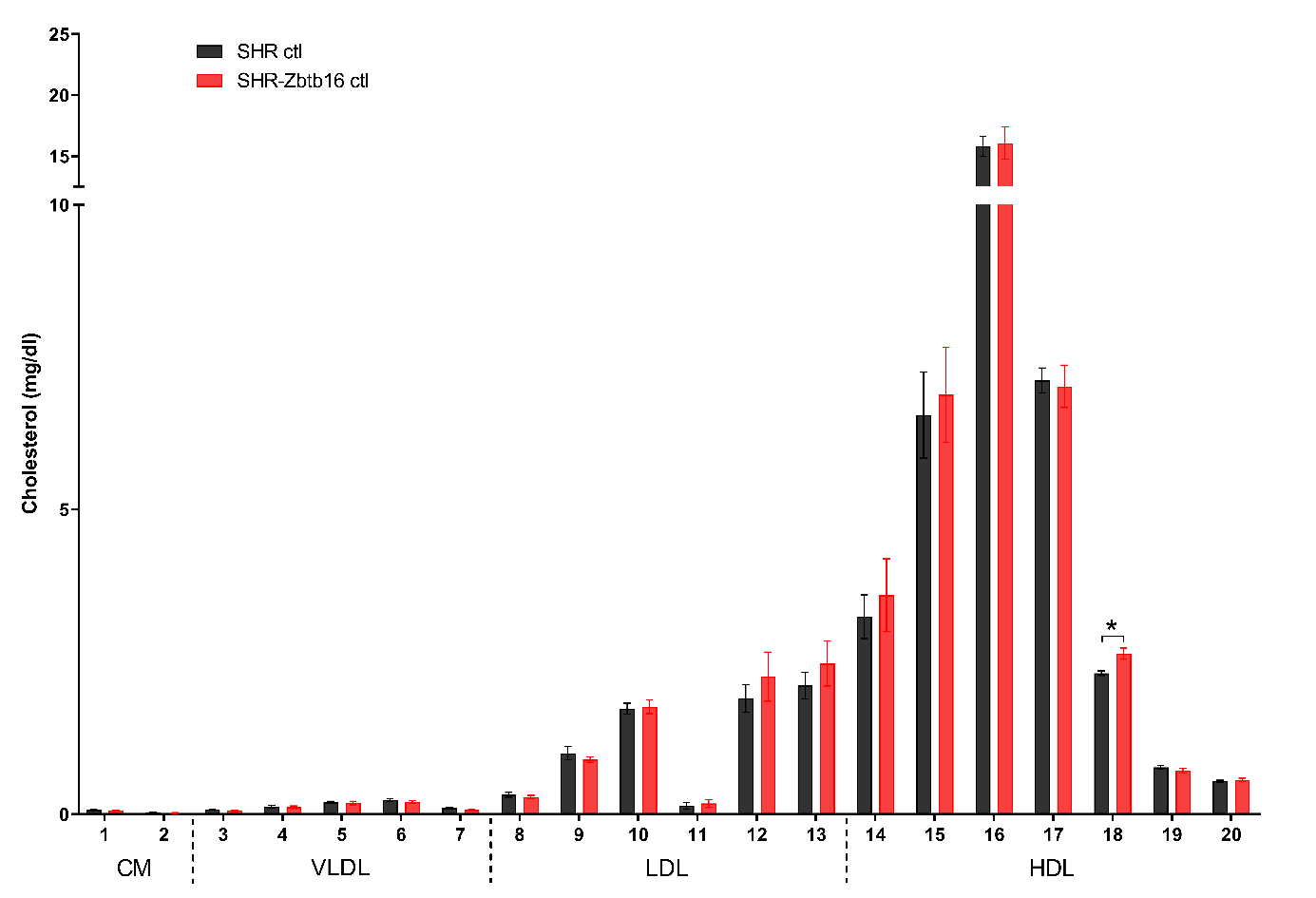


**Supplementary Figure 3:** The cholesterol content in 20 lipoprotein subfractions in SHR female rats (black bars) and SHR-*Zbtb16* female rats (red bars) before pregnancy. Within the graph, the significance levels of pairwise comparisons between strains by post-hoc Fisher's test of the two-way ANOVA with STRAIN and DIET as major factors are indicated as follows: * p<0.05. The allocation of individual lipoprotein subfractions to major lipoprotein classes is shown in order of particle's decreasing size from left to right. CM-chylomicron, VLDL-very low-density lipoprotein, LDL-low density lipoprotein, HDL-high density lipoprotein.
